# Life is unfair, and so are racing sports: some athletes can randomly benefit from alerting effects due to inconsistent starting procedures

**DOI:** 10.3389/fpsyg.2015.01618

**Published:** 2015-10-28

**Authors:** Edwin S. Dalmaijer, Beorn G. Nijenhuis, Stefan Van der Stigchel

**Affiliations:** ^1^Department of Experimental Psychology, University of Oxford, Oxford, UK; ^2^Department of Experimental Psychology, Utrecht University, Utrecht, Netherlands

**Keywords:** alerting, temporal expectancy, foreperiod, racing, sports

## Abstract

The Olympics are the world’s largest sporting events, attracting billions of viewers worldwide. Important parts are racing sports, such as running, swimming and speed skating. In these sports, athletes compete against each other in different heats to determine who wins the gold, or who is granted a place in the final. Of course, the gold goes to whoever is the most talented and has trained the hardest. Or does it? Here we argue that subtle differences between athletes’ starts can bias the competition, and demonstrate this in the results of speed skating at the 2010 Winter Olympics. This bias could be removed by simple alterations to current starting procedures. The proposed change would greatly improve racing sport fairness, which currently suffers from an injustice that disadvantages not only athletes, but entire nations rooting for them.

## Starting Regulations

Regulators try to make sports fair: cheating is punished, and procedures are as similar as possible between athletes. However, sometimes small procedural inconsistencies are overlooked. Such an inconsistency can be found in the starting procedure of racing sports, where a referee alerts participants of the imminent start with a warning cue (“*Ready?*”), and fires a starting gun after a *variable* interval. The variability of this interval is dependent on how long athletes take to assume their starting positions (swimming, rule SW 4.1, Fédération Internationale de Natation, 2013; and running, rule 111-8, UK Athletics, 2014), sometimes with the addition of temporal jitter (speed skating, rule E-255, International Skating Union, 2014).

Almost every detail of racing sports has been subject to scientific investigation, including ice (De Koning et al., 1992; Lozowski et al., 2013) and air friction (Van Ingen Schenau, 1982), skates (De Koning et al., 2000), suits (Sætran and Oggiano, 2008), and even its innovative history (Versluis, 2005). In fact, science has been a driving force behind world records (De Koning, 2010). Importantly, the starting procedure has received much attention. Research has defined what starting parameters separate elite from sub-elite sprinters (Fortier et al., 2005), the effects of different starting positions, timing devices, and false starts in running have been well described (Haugen et al., 2012, 2013), and even the effects of starting with the left or right foot are known (Eikenberry et al., 2008). Despite all this research, the aforementioned variability in starting procedures has not been under rigorous scientific appraisal. Here, we argue that subtle variabilities in the timing of starting procedures are an important issue, as they can bias race times in Olympic competitions.

## Alerting Effects in Experimental Psychology

The starting procedure in racing sports closely resembles a classical experiment, where participants receive an alerting cue before having to respond to a target stimulus. The cue is a general, non-spatial signal that precedes the target stimulus by a variable interval. In the lab, participants are quicker (Posner and Boies, 1971; Adams and Lambos, 1986) and more precise (Klein and Kerr, 1974) to respond after an optimal interval duration of around 500 ms, and are progressively slower and less precise after longer durations.

It is important to note that alerting is dissociable from selective attention orienting (Weinbach and Henik, 2012a, 2013). Selective attention for stimuli occurring at *specific* instants in time can also reduce response times and increase accuracy (Coull and Nobre, 1998). Although these effects resemble the effects of general alerting (e.g., in blocked foreperiod experiments), they originate from specific temporal expectancies (Niemi and Näätänen, 1981; Nobre, 2001; Nobre et al., 2007), whereas alerting effects have been attributed to a short and non-specific increase in arousal (Posner and Boies, 1971). In addition, alerting and selective temporal attention are behaviourally distinct (but additive; Weinbach and Henik, 2012b), and rely on separate brain networks (Raz and Buhle, 2006).

In most experimental designs, the effects of alerting and temporal expectancy cannot be dissociated, and can even be confounded (Weinbach and Henik, 2012a). However, it is possible to isolate each mechanism in the lab by using a clever design (Lawrence and Klein, 2013). Interestingly, evidence of this dissociation can already be found in classic research: Sanders (1975) used cues of different intensities in a blocked foreperiod experiment, where temporal expectancy was induced by using the same foreperiod (time between cue and target) within a block. In this design, more intense cues were thought to induce a higher level of alertness, whereas temporal expectancy was thought to deteriorate with longer foreperiod durations due to accumulation of noise (Niemi and Näätänen, 1981). Sanders (1975) found the expected effects of temporal expectancy (lower reaction times for lower foreperiods), and of alerting (lower reaction times for more intense cues). Importantly, these differential (but additive) effects were present at foreperiods of 1 s and of 5 s, disputing the belief that alerting only occurs on short time scales.

In sum, alerting and temporal expectancy are qualitatively different mechanisms, although their time courses can overlap (Weinbach and Henik, 2012a). Temporal expectancy produces a benefit for a specific point in time, for example the most probable time between cue and target (Thomaschke et al., 2011). Alerting, however, refers to a general boost of arousal following a cue. Alerting can be modulated by a cue’s intensity, and can last for several seconds (e.g., as seen in Sanders, 1975).

## Alerting Effects Bias Racing Sports

If all athletes were subject to the same ready-start interval, no bias would exist. However, most racing events consist of multiple races, and thus athletes are subjected to randomly different ready-start intervals. In running and swimming, athletes’ times are compared between heats, and only the top few advance to the next round. Considering the alerting benefit for shorter ready-start intervals, athletes starting in a heat with a shorter ready-start interval have a higher chance of reaching the final.

In speed skating, the potential consequences are worse. A competition consists of many races, with two athletes competing at a time. Individuals’ times are compared between races, and the lowest total time wins. The result is that athletes who start with the shortest ready-start intervals have a better chance of winning the gold.

One could expect that other factors, such as talent and training, strongly outweigh the theoretical benefit of starting with a shorter ready-start interval. This is a fair point, considering the vast differences between the environments of a sports event and a psychological laboratory. Therefore, we examined the finishing times for the 500 m speed skating competition at the 2010 Winter Olympics, in relation to millisecond-accurate ready-start intervals obtained directly from the audio trace of the television broadcast. The audio traces were analyzed by visual inspection in Audacity, and the ready-start intervals were defined as the time between the onset of the referee saying “Ready?” and the onset of the starting shot.

**FIGURE 1:**
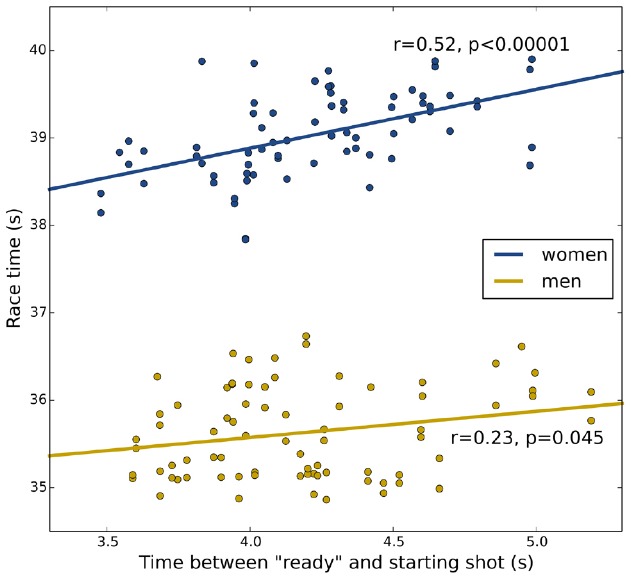
**Relation between ready-start intervals and race outcome.** In the 500 m speed skating competition of the 2010 Winter Olympics, there was a significant correlation of ready-start interval and both men’s (yellow) and women’s (blue) race times. The longer the interval between the referee’s “*Ready*” cue and the starting shot was, the worse athletes performed.

There are statistically significant correlations between ready-start intervals and race times (Figure 1), explaining 5% of the variance in men’s times and 27% of the variance in women’s times. The magnitudes of these coefficients of determination indicate that the theoretical alerting benefit we describe has a very real impact on athlete’s race times. In our sample, one extra second of ready-start interval raised the finishing time by an average 672 ms in women, and 299 ms in men (assuming a linear relation between ready-start interval and finishing time). In high-level speed-skating competitions, this can mean the difference between first and fifth place.

We only excluded races in which athletes fell or severely lost their balance (3 out of 148 individual races). False starts occurred in only six of the men’s races, and only two of the women’s races. This scarcity makes it very unlikely that falls or false starts drive the effect of ready-start interval on race times. If anything, the presence of false starts seems to obscure it.

In our view, the correlation between ready-start interval duration and race time is driven by alertness: athletes starting with a shorter ready-start interval are more alert at the time of the starting shot, and thus quicker to finish. Even if one could conceive another explanation, it would not render the correlation invalid. It is clear that something is amiss, and whether this is really due to the “ready” cue’s alerting effect is an important question for future research.

## A Potential Solution

The fairness of any racing sports’ starting procedure could be improved by introducing an extra step, and removing temporal variability. In our remedied start, a referee signals athletes to get ready (“*Get Set*”), and explicitly cues the impeding start only *after* everyone has assumed position (“*Ready*”). After a fixed time, the starting shot should sound. Ideally, to prevent human timing error, the referee could simply press a button after all athletes have assumed their starting positions. This would activate a computerized system that produces both the “*Ready*” cue and the starting shot, separated by a fixed interval.

Although using a fixed interval will equalize *general* alerting benefits, it will not remove the effects of *selective* temporal expectancy. In fact, it will increase athletes’ ability to anticipate the starting shot. Importantly, incorporating a fixed ready-start interval in official rules and regulations will allow athletes to train their response times for that specific interval. This would make an athlete’s temporal attention an individual quality that contributes to their likelihood to win. In our view, this would be fairer than the current starting procedure, which is essentially a lottery.

## Conclusion

In racing sports, there are subtle starting differences between races within competitions. Athletes start after a randomly varying interval between a cue to get ready and the starting shot. The shorter this interval is, the more alert athletes are, and the quicker they can respond to the starting shot. This randomly benefits some athletes over others, and significantly biases race times. The issue could be resolved by introducing a three-step starting procedure with fixed timing.

### Conflict of Interest Statement

The authors declare that the research was conducted in the absence of any commercial or financial relationships that could be construed as a potential conflict of interest.
